# Development and Optimisation of an Industrial Waste-Based Additive for Improving Concrete Performance

**DOI:** 10.3390/ma19091698

**Published:** 2026-04-23

**Authors:** Rauan Lukpanov, Duman Dyussembinov, Aliya Altynbekova, Serik Yenkebayev, Lyailya Kabdyrova, Denis Tsygulyov

**Affiliations:** 1 Solid Research Group, LLP, Astana 010008, Kazakhstan; rauan_82@mail.ru (R.L.); dusembinov@mail.ru (D.D.); enkebaevserik@gmail.com (S.Y.); leila_0781@mail.ru (L.K.); denis_riza_72@mail.ru (D.T.); 2 Department of Technology of Industrial and Civil Engineering, L.N. Gumilyov Eurasian National University, Astana 010008, Kazakhstan; 3 Department of Construction, L.N. Gumilyov Eurasian National University, Astana 010008, Kazakhstan

**Keywords:** concrete, modified additive, soapstock, phosphogypsum, microsilica, post-alcohol bard, compressive strength, water absorption, frost resistance

## Abstract

This study investigates the development and optimization of a multi-component modifying additive based on industrial waste for improving the mechanical and durability properties of concrete. The additive consists of microsilica (Ms), phosphogypsum (PhG), soapstock (Sp), and post-alcohol bard (PaB), and its performance was evaluated using a staged experimental approach. The results showed that the optimal content of microsilica is 20% of the cement mass; the optimal content of phosphogypsum is 15% of the combined mass of the cement and microsilica; the optimal content of soapstock is 10% of the total mass of the cement, microsilica, and phosphogypsum; and the optimal post-alcohol bard is 5% of the water mass. At these concentrations, the compressive strength increased by up to 28.3% compared to the reference sample. Soapstock significantly reduced water absorption (up to 36.8%) and improved freeze–thaw resistance due to the hydrophobization of the cement matrix. However, excessive soapstock content led to a reduction in strength. The addition of post-alcohol bard provided a plasticizing effect and reduced water absorption, with the optimal concentration for strength being 2.5%, while the highest freeze–thaw resistance was observed at 5%. The combined effect of the components resulted in the formation of a denser microstructure and improved durability of concrete. These findings demonstrate the effectiveness of industrial waste-based additives in enhancing concrete performance and durability, contributing to sustainable material development.

## 1. Introduction

Modern construction increasingly requires high-performance building materials, especially concrete. Despite its widespread use, traditional concrete has several drawbacks, such as high water absorption, limited frost resistance, and a tendency to crack under thermal or drying stresses [1]. Additionally, cement production remains one of the most resource-intensive and environmentally damaging industrial processes, accounting for roughly 8% of global CO_2_ emissions [2].

To address these issues, researchers have investigated developing modified concrete mixtures that use industrial by-products, recycled materials, and chemical additives to improve physical and mechanical properties while lowering the environmental impact of concrete production [3,4,5]. However, most studies focus on single additives, while the combined effects of multiple components are not well studied. This gap highlights the scientific problem of the current research.

Microsilica (Ms), a by-product of ferrosilicon alloy production, exhibits high pozzolanic activity, significantly reducing porosity and increasing concrete strength, as demonstrated in [6]. It also enhances impermeability and long-term durability, particularly in chemically aggressive environments [7].

Phosphogypsum (PhG), a by-product of phosphate fertilizer production, has attracted significant research interest as a potential partial cement replacement due to its abundant availability and environmental benefits of valorizing industrial waste. Studies [8,9] have shown that it enhances sulfate resistance, reduces the heat of hydration, and promotes the formation of a more stable microstructure. However, the presence of impurities (e.g., fluorides and phosphates) requires careful treatment and dosage control [10].

Recent research has shown that phosphogypsum can be effectively recycled and incorporated into cementitious systems. For example, comprehensive reviews highlight various pretreatment and application strategies for phosphogypsum in cementitious materials, demonstrating improvements in hydration and performance properties [11]. Phosphogypsum-based ultra-low-basicity cementitious materials with improved mechanical performance have been developed [12]. Supersulfated cements with calcined phosphogypsum exhibit favorable hydration behavior and strength development [13]. Studies also report the strength characteristics and mechanisms of cement-stabilized phosphogypsum blends under environmental cycling [14], as well as optimized formulations and hydration mechanisms that contribute to performance enhancement [15].

Soapstock (Sp), a by-product of the oil refining industry, is used as an air-entraining and hydrophobic agent. Studies [16,17] indicate that soapstock improves the frost resistance of concrete by reducing capillary absorption and promoting the formation of closed air voids. Its economic efficiency has also been demonstrated in industrial applications [18].

Post-alcohol bard (PaB), a residue from ethanol production, contains organic plasticizing compounds that increase the workability of concrete mixtures. Studies [19,20] have shown that it reduces the water–cement ratio and improves early-age strength. However, excessive amounts may delay cement hydration [21].

The selection of the four components was not arbitrary but based on their complementary physicochemical functions within the cement system. Microsilica was used as a well-established pozzolanic additive to enhance strength and reduce porosity through the formation of additional C–S–H phases. However, its role in this study is not considered as novel individually, but as a reference mineral component forming the base of the modifying system. Phosphogypsum was introduced as a source of calcium sulfate to regulate hydration processes and modify the mineralogical balance of the binder. Soapstock and post-alcohol bard were selected as organic components providing hydrophobic and plasticizing effects, respectively.

Thus, the combination of mineral (Ms, PhG) and organic (Sp, PaB) components allows the formation of a multifunctional system, in which strength development, pore structure modification, and durability characteristics are controlled simultaneously. The staged experimental design was adopted to isolate the individual contribution of each component and to avoid overlapping effects, enabling the identification of optimal concentrations within the combined system.

Despite these promising findings, comprehensive studies on the combined use of these additives remain limited. Critical literature reviews [22,23,24,25,26,27] emphasize the lack of data on the synergistic effects arising from the interaction of organic and mineral additives within a single modifying system.

In the regional context, significant volumes of industrial waste, including microsilica, phosphogypsum, soapstock, and post-alcohol bard, are accumulated, and their utilisation represents an important environmental and technological challenge. Their incorporation into concrete mixtures not only reduces environmental impact but also provides an additional raw material base for the construction industry.

The scientific novelty of this study lies in the rational selection and combination of heterogeneous waste materials with different mechanisms of action. The proposed system enables a coordinated influence on particle packing, hydration processes, and rheological behaviour, resulting in a comprehensive improvement in concrete performance.

The main objective of this study is to design and experimentally validate a multifunctional additive that enhances the physical and mechanical properties of concrete mixtures while reducing the environmental impact of the construction industry.

The results have practical significance for construction in continental and sharply continental climates, where frost resistance, low permeability, and structural stability under temperature fluctuations are critical. The proposed additive also promotes the recycling of industrial waste, reduces concrete production costs, and improves the durability of concrete structures.

## 2. Materials and Methods

The proposed additive is a composite material derived from industrial waste and consists of both solid and liquid components. The solid component (Component 1, C1) is a dry mixture of microsilica, phosphogypsum, and neutralized soapstock, whereas the liquid component (Component 2, C2) consists of post-alcohol bard.

Each component is introduced independently into the concrete mixture, replacing standard constituents by mass: C1 replaces cement, while C2 replaces water. However, determining the quantitative proportions of these components within the dry additive and in the prepared concrete mix, including the liquid phase, is a key objective of this study. Defining the optimal composition of the modified additive requires a series of sequential investigations to assess the effect of each component on the performance characteristics of the cement–sand mixture:

Stage 1: Evaluation of the effect of microsilica at varying proportions relative to the replaced cement.

Stage 2: Evaluation of the effect of phosphogypsum at varying proportions relative to the binder (cement and the previously determined optimal microsilica content).

Stage 3: Evaluation of the effect of soapstock at varying proportions relative to the binder (cement and the previously determined optimal contents of microsilica and phosphogypsum).

Stage 4: Evaluation of the effect of post-alcohol bard at varying proportions relative to the replaced water (water–cement ratio).

Upon completion of these studies, the optimal component concentrations are determined, and the composition of the modified additive is adjusted accordingly by mass.

The staged sequence of the study is based on the following rationale: the incorporation of microsilica into the cement–sand mixture alters the mineral composition through the introduction of fine particles. Therefore, the addition of phosphogypsum, which contains calcium capable of reacting with silica, should not precede the incorporation of microsilica. The incorporation of soapstock and post-alcohol bard represents parallel processes that are independent of each other but contribute to material modification, specifically by enhancing durability (due to the hydrophobization of concrete) and improving strength (through a plasticizing effect). The sequence of component introduction was determined by their hierarchical roles in the cementitious system. Mineral components were evaluated first to establish the base microstructure and hydration regime, followed by organic modifiers affecting pore structure and rheological behaviour. This approach minimises overlapping effects and ensures a clearer interpretation of individual contributions. Table 1 presents the variable compositions of the mixtures at each stage of the study.

During the experimental program, the concrete mixtures were designed in accordance with the requirements of PN-EN 206 [29], which specifies the composition, performance, and conformity criteria for concrete. During the experimental program, the total binder content in all mixtures was kept constant at 500 g. The binder consisted of cement, microsilica, phosphogypsum, soapstock, and NaOH, depending on the experimental stage. In addition, the total liquid content was maintained at 200 g, with stillage partially replacing the mixing water. This approach allowed for the evaluation of the effects of the additive components without altering the overall binder or liquid content of the mixture.

The materials used in this study were as follows:

Cement: Portland cement of grade M400, classified as CEM I 42.5 N, was sourced from «Kokshe-Cement» (Kokshetau, Kazakhstan). The setting times were as follows: initial set 2 h 45 min; final set 6 h 10 min. The chemical composition of the cement is presented in Table 2.

Fine and Coarse Aggregates: Sand (particle size < 2.5 mm) from the Ilyinovskoye deposit was used as the fine aggregate, while crushed stone (5–20 mm) from the Tastak deposit served as the coarse aggregate.

The composite additive consisted of four components: microsilica (Ms), phosphogypsum (PhG), soapstock (Sp), and post-alcohol bard (PaB). The materials were used in the following proportions: microsilica was added at 10%, 15%, 20%, and 25% by mass relative to the cement; phosphogypsum was used at 10%, 15%, 20%, and 25% by mass relative to the combined mass of microsilica and cement; soapstock combined with sodium hydroxide was incorporated at ratios of 5.0%, 7.5%, 10.0%, and 12.5% by mass relative to microsilica, phosphogypsum, and cement, with the sodium hydroxide content maintained at 1% by mass of the soapstock; post-alcohol bard was added at 2.5%, 5.0%, 7.5%, and 10% by mass relative to water. The characteristics of the additive components are presented in Table 3. The additive components (microsilica, phosphogypsum, soapstock, and post-alcohol bard) were used in their as-received condition without additional purification. This approach reflects practical industrial conditions, where further processing of waste materials would increase production costs and reduce economic feasibility. The study is focused on evaluating the combined performance of the selected components within the cementitious system. The physicochemical effects of the additives were therefore assessed at the level of the hardened composite material after hydration.

**Figure 1:**
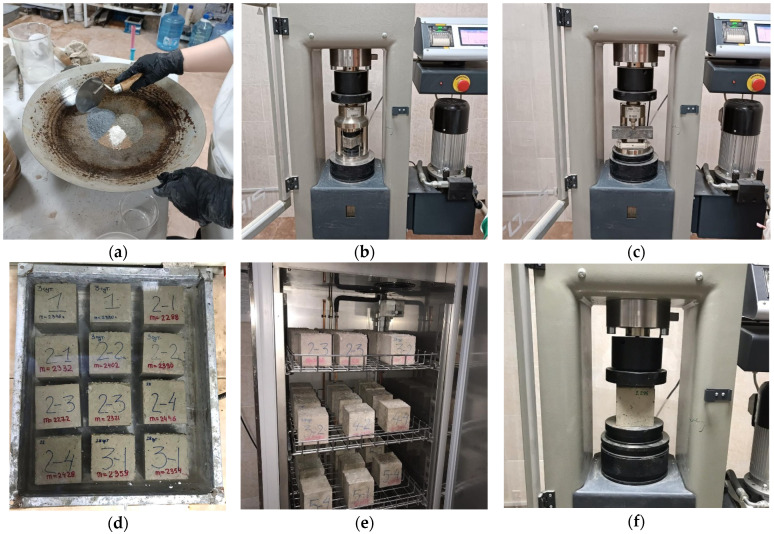
Testing Process: (**a**) component preparation; (**b**) compressive strength (beam samples); (**c**) flexural strength; (**d**) water absorption; (**e**,**f**) frost resistance and compressive strength (cubic samples).

The main research methods include (Figure 1):

– Evaluation of the elemental composition of the samples;
– Measurement of the compressive and flexural strength of beam samples;
– Determination of the water absorption of the samples;
– Assessment of the frost resistance of the samples.

The composition of the samples was analyzed using EDX to determine the elemental composition and to assess the effects of the additive components on the overall composition (Figure 1a). In this experiment, the analysis was performed using SEM micrographs obtained with a tabletop TM4000Plus scanning electron microscope (Hitachi, Tokyo, Japan), operating at an accelerating voltage of up to 20 kV, with a magnification range of 10× to 25,000× and a resolution of 3.5 nm for W. Evaluating the changes in composition resulting from the addition of different components is necessary to assess the performance of the additive and determine its potential suitability for improving the physicochemical properties of cement.

The compressive and flexural strength of the samples were measured using a Press Automatic Pilot Controls machine (Milan, Italy) with a maximum compressive load of 500 kN (50 tons) at 7, 14, and 28 days, in accordance with GOST 310.4 [31] and EN 196-1:2016 [32] (Figure 1b,c). Comparisons of the strength of samples with varying compositions were conducted to determine the optimal composition of the modified additive and evaluate its performance. Additionally, comparing the strength parameters of samples with and without the additive provides insight into the effects of the additive components on concrete modification and the resulting improvements in strength.

The water absorption of the concrete samples was determined in accordance with GOST 12730.3-2020 [33] and ASTM C642-21 [34] (Figure 1d). The samples were submerged in water at a depth of 60 mm and a temperature of 21 °C, with their weight measured every 24 h until a constant mass was reached (mass variation not exceeding 0.1% over 120 min). To dry the samples, they were exposed to a temperature of 100 ± 5 °C for at least 24 h. This soaking procedure ensured complete water absorption. Comparing the water absorption of the samples provides insight into the operational suitability of concrete containing the modified additive, particularly regarding its service life. In addition, the hydrophobic properties of the material were evaluated as an indicator of its resistance to water-induced damage during service, which also correlates with improved frost resistance as observed in the frost resistance tests. Frost resistance of the concrete samples was assessed using a Climate Chamber 10-D1429/A Controls (Milan, Italy) (550 L volume) in accordance with GOST 10060 [35] and BS EN 12390-Part 9 [36] (Figure 1e,f). Comparison of frost resistance indicators across different concrete types provides further evaluation of material durability and operational performance. The sequence of cyclic freezing and thawing was designed to optimize testing: as the number of cycles increased, the duration of control measurements was reduced (from 50 to 25 cycles). Strength and mass were measured at 50, 100, 150, 175, 200, 225, and 250 cycles. Changes in mass and strength were analyzed to assess the material’s resistance to cyclic temperature effects and its overall durability.

The evaluation criterion for Stage 1 was the compressive and flexural strength of beam samples (cement strength) with dimensions of 160 × 40 × 40 mm^3^. This criterion was selected to assess the potential influence of microsilica as an active ultrafine mineral on the strength characteristics of concrete. For Stage 2, the evaluation criteria included water absorption and frost resistance of standard cubic samples measuring 100 × 100 × 100 mm^3^. These criteria were established based on the presence of fatty acids in soapstock, which contribute to the volumetric hydrophobization of concrete. In Stage 3, additional evaluation criteria included monitoring the strength of beam samples at 28 days of age to assess the development of strength characteristics observed in Stages 1 and 2, along with analysis of statistical data from control cubic samples during frost resistance testing. Stage 3 criteria also included water absorption and frost resistance of standard cubic samples (100 × 100 × 100 mm^3^), determined based on the plasticizing effect of post-alcohol bard, which contains casein that reduces internal stresses in concrete. In Stage 4, the evaluation criteria included the strength of cement–sand beam samples and the control of cubic concrete samples during frost resistance assessment. Each composition was tested using three replicates to ensure accuracy and repeatability.

**Figure 2:**
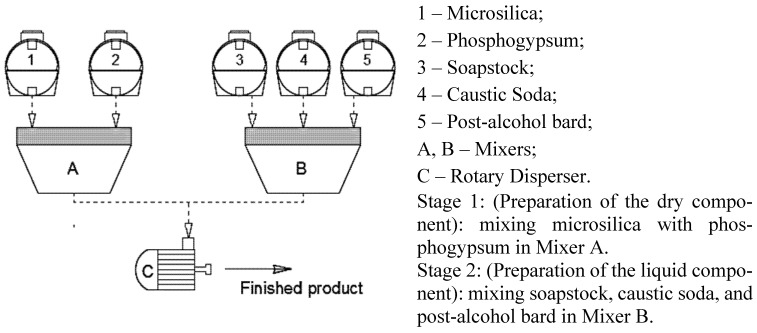
Additive Production Technology.

Figure 2 illustrates the technological workflow for producing the modified additive. The process is divided into two sequential stages. In the first stage, the dry component is prepared by grinding, drying, and blending microsilica and phosphogypsum. Grinding ensures the formation of a homogeneous ultrafine mixture, which is crucial for enhancing the activity of the components during concrete hydration. Drying allows for precise mass measurement and removes any residual moisture in the additive. In the second stage, the liquid component is prepared by combining soapstock with post-alcohol bard, followed by neutralization to adjust the acidity.

## 3. Results

### 3.1. Results of EDX Analysis

**Figure 3:**
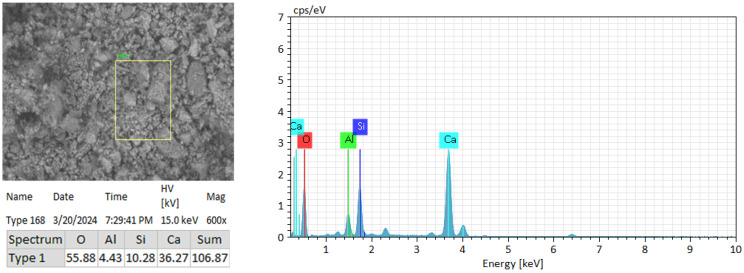
Results of EDX analysis of the reference sample.

**Figure 4:**
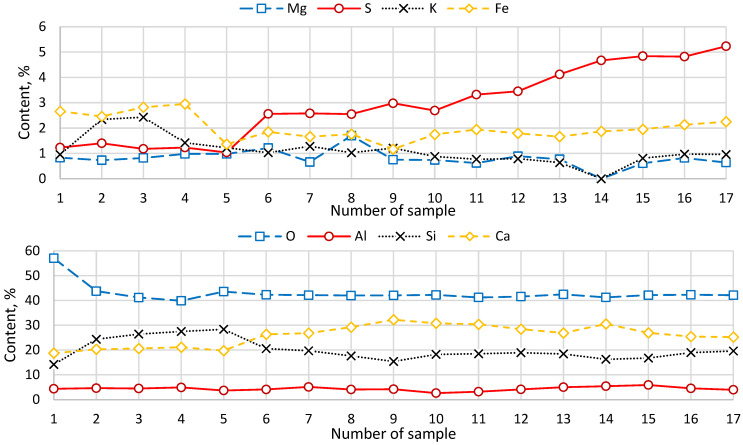
Results of EDX analysis of samples with additives.

Figure 3 presents the results of EDX analysis of the reference samples, which served as the basis for the elemental analysis (expressed as weight, %). Figure 4 illustrates the changes in elemental composition resulting from the addition of the additive components (expressed as weight, %).

The x-axis represents the sample number: 1—reference sample; 2–5—addition of microsilica at 10–25%; 6–9—addition of phosphogypsum at 10–25%; 10–13—addition of soapstock at 5.0–12.5%; and 14–17—addition of post-alcohol bard at 2.5–10.0%. According to the EDX analysis of the reference sample (unmodified cement binder), a high oxygen content of 57% was observed, indicating the presence of compounds that do not participate in cement hydration or in the formation of the cement paste structure. When calculating the required amount of oxides for the hydration process, considering all chemical components, the oxygen content should not exceed 14%.

The addition of microsilica to the cement binder resulted in a natural increase in the silica content of the samples. For microsilica additions ranging from 0 to 20%, the oxygen content decreased from 57.05% to 39.89%. For complete hydration of cement, the required oxygen content is approximately 21%; therefore, these results suggest that microsilica contributes to a reduction in the proportion of compounds not involved in hydration and to improved cement paste formation. When the microsilica content was further increased to 25%, oxygen increased to 43.6%, which negatively affected concrete quality, as confirmed by the physical and mechanical properties of the samples. The contents of Mg, Al, S, K, and Fe remained essentially unchanged, with variations within the statistical error.

When phosphogypsum was added to a cement binder containing 25% microsilica, an increase in Ca and a relative decrease in Si (as a percentage of total composition) were observed. The increase in Ca and S (compared to samples without phosphogypsum) is attributed to the presence of up to 97% CaSO_4_ in phosphogypsum. The absolute silicon content remained constant; the percentage decrease is due to the increase in total Ca. Oxygen content remained within the optimum range, with a median of 42%. Other elements, including Mg, Al, K, and Fe, showed no significant change.

The addition of soapstock to the cement binder containing 25% microsilica and 25% phosphogypsum resulted in increases in Al and S. The NaOH present in soapstock increased the alkalinity, thereby enhancing the activity of portlandite with respect to Al. Absolute Ca content remained constant; the decrease in its percentage is due to the relative increase in total Al and S. Oxygen content remained within the optimum range, with a median of 42%. The contents of Mg, Si, K, and Fe remained unchanged.

When post-alcohol bard was added to a cement binder containing 25% microsilica, 20% phosphogypsum, and 12.5% soapstock, an increase in S was observed. The elevated sulfur content (more than 4.67%) indicates potential bulk gypsumization during cement hydration, contributing to increased compressive and flexural strength. Absolute Ca and Si contents remained constant; the percentage decrease is due to the increase in total S. The decrease in Al percentage is associated with the primary interaction of Ca with portlandite. Oxygen content remained within the optimum range, with a median of 42%. The amounts of Mg, K, and Fe remained unchanged.

### 3.2. Results of Stage 1 Testing

**Figure 5:**
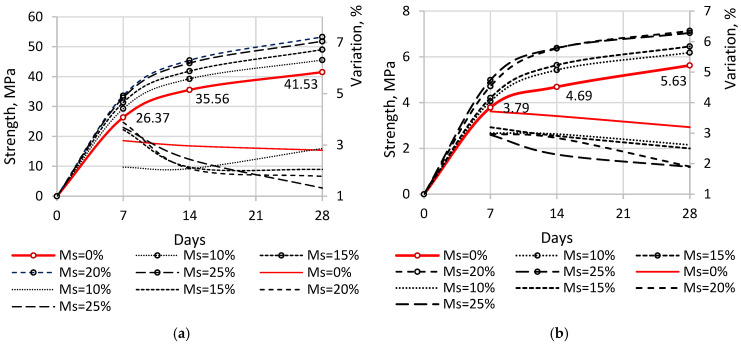
Comparative Strength Diagrams: (**a**) compressive strength; (**b**) flexural strength.

**Figure 6:**
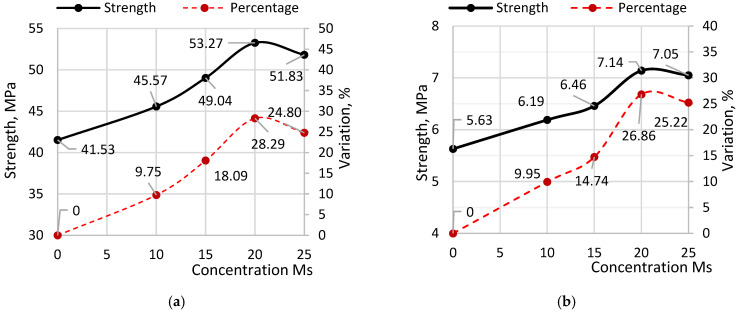
Strength Variation with Microsilica Content: (**a**) compressive strength; (**b**) flexural strength.

Figure 5 and Figure 6 present comparison charts of the measured strength values at different ages for samples with varying microsilica (Ms) concentrations, along with their corresponding coefficients of variation for compressive and flexural strength.

The compressive strength of the reference sample (without additive) for beam samples averaged as follows: 26.37 MPa at 7 days, 35.55 MPa at 14 days, and 41.53 MPa at 28 days. The corresponding flexural strength values were 3.59 MPa at 7 days, 4.78 MPa at 14 days, and 5.42 MPa at 28 days. For samples containing 10% microsilica (Ms), compressive strength was 29.27 MPa at 7 days (+11.0% compared to the reference), 39.26 MPa at 14 days (+10.4%), and 41.53 MPa at 28 days (+9.7%). Samples with 15% Ms exhibited compressive strengths of 31.35 MPa at 7 days (+18.9%), 41.84 MPa at 14 days (+17.7%), and 49.04 MPa at 28 days (+18.1%). For 20% Ms, compressive strength increased to 33.63 MPa at 7 days (+27.5%), 45.47 MPa at 14 days (+27.9%), and 53.27 MPa at 28 days (+28.3%). Samples with 25% Ms showed compressive strengths of 32.95 MPa at 7 days (+24.9%), 44.52 MPa at 14 days (+25.2%), and 51.83 MPa at 28 days (+24.8%).

The flexural strength of samples with 10% Ms was 4.07 MPa at 7 days (+7.1%), 5.43 MPa at 14 days (+15.8%), and 6.19 MPa at 28 days (+9.9%). Samples with 15% Ms reached 4.21 MPa at 7 days (+11.1%), 5.64 MPa at 14 days (+20.3%), and 6.46 MPa at 28 days (+14.7%). For 20% Ms, flexural strength was 4.72 MPa at 7 days (+24.6%), 6.37 MPa at 14 days (+35.8%), and 7.14 MPa at 28 days (+26.8%). Samples with 25% Ms showed flexural strengths of 4.99 MPa at 7 days (+31.6%), 6.37 MPa at 14 days (+36.2%), and 7.05 MPa at 28 days (+25.2%).

In addition to strength analysis, the effect of microsilica (Ms) on the flowability of the cement–sand mixture was evaluated using the slump flow method in accordance with EN 12350-5 [37]. Flowability is a critical property for ensuring proper compaction and uniformity of concrete, and the presence of ultrafine silica particles significantly influences the rheology of fresh mixes.

The reference mixture (without Ms) exhibited a slump flow diameter of 188 mm. With the addition of 10% Ms, the flowability decreased to 173 mm; at 15% Ms, it was 162 mm; at 20% Ms, 154 mm; and at 25% Ms, 144 mm. This progressive reduction in slump flow indicates a clear decrease in workability due to the high specific surface area and water demand of microsilica, which increases internal friction and reduces the availability of free water in the mix.

Although compressive and flexural strength improved with Ms content up to 20%, the corresponding loss of flowability may impede proper compaction in practical applications, potentially affecting the homogeneity and porosity of the hardened concrete matrix. Therefore, optimization of Ms content should balance both mechanical and rheological performance.

The coefficients of variation generally indicate a strong correlation among individual strength measurements, showing an overall trend toward stabilization as the samples gain strength. For compressive strength, the coefficients of variation ranged from 1.32% to 3.88%, while for flexural strength, they ranged from 1.89% to 3.71%. To verify the statistical significance of the observed improvements, a *t*-test was performed for the 28-day strength values. The compressive and flexural strength of mixtures containing 20% microsilica were found to be significantly higher than those of the reference mixture (*p* < 0.001), confirming that the observed differences are not due to experimental variability.

Figure 6 illustrates the changes in strength values as a function of microsilica content in the samples.

According to the diagrams, the maximum increase in both compressive and flexural strength occurs at a microsilica content of 20% relative to cement. Peak compressive strength reached 53.27 MPa, representing a 28.29% increase over the reference sample without additives. Peak flexural strength reached 7.14 MPa, exceeding the reference sample by 26.82%. When microsilica content exceeds 20%, a reduction in strength is observed for both compressive and flexural measurements. This decline may be attributed to the high silicon content of microsilica, which is approximately 90% or higher. The addition of microsilica enhances the pozzolanic reaction by consuming portlandite and forming additional calcium silicate hydrate (C–S–H), which contributes to strength development. However, excessive microsilica may reduce the efficiency of this process due to limited availability of portlandite or insufficient curing time required for complete pozzolanic reaction.

### 3.3. Results of Stage 2 Testing

**Figure 7:**
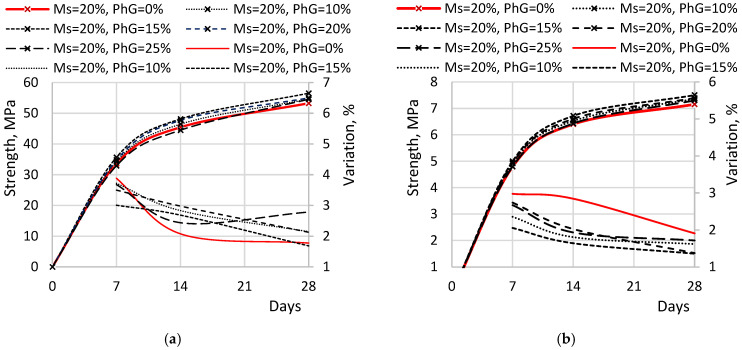
Comparative Diagrams of Strength Indicators: (**a**) compressive strength; (**b**) flexural strength.

Figure 7 presents comparison diagrams of the measured compressive and flexural strength values at different curing ages for samples with varying phosphogypsum (PhG) concentrations, along with their corresponding coefficients of variation. The assessment of strength variations for PhG contents ranging from 10% to 25% was conducted relative to the average strength of samples containing the previously determined optimal microsilica content of 20%.

The compressive strength for samples with a PhG content of 10% was: at 7 days of age, 34.26 MPa, which exceeds the strength of $M_{s}^{20}$ by 1.9% and the reference sample by 29.9%; at 14 days, the strength is 46.46 MPa, exceeding $M_{s}^{20}$ by 2.2% and the reference sample by 30.7%; at 28 days, it is 54.62 MPa, exceeding $M_{s}^{20}$ by 2.5% and the reference sample by 31.5%. Samples with a PhG content of 15% showed the following strength indicators: at 7 days, 35.69 MPa, which exceeds $M_{s}^{20}$ by 6.1% and the reference sample by 35.4%; at 14 days, the strength is 48.17 MPa, exceeding $M_{s}^{20}$ by 5.9% and the reference sample by 35.5%; at 28 days, it is 56.54 MPa, exceeding $M_{s}^{20}$ by 6.1% and the reference sample by 36.1%. For samples with a PhG content of 20%, the strength was: at 7 days, 34.8 MPa, which exceeds $M_{s}^{20}$ by 3.5% and the reference sample by 31.9%; at 14 days, the strength is 47.71 MPa, exceeding $M_{s}^{20}$ by 4.9% and the reference sample by 34.2%; at 28 days, it is 55.01 MPa, exceeding $M_{s}^{20}$ by 3.3% and the reference sample by 32.5%. For samples with a PhG content of 25%: at 7 days, 32.95 MPa, which is 2.1% lower than $M_{s}^{20}$, but 24.9% higher than the reference sample; at 14 days, the strength is 44.52 MPa, which is also 2.1% lower than $M_{s}^{20}$, but higher than the reference sample by 25.2%; at 28 days, it is 54.57 MPa, which exceeds $M_{s}^{20}$ by 2.4%, while the strength of the reference sample exceeds it by 31.4%.

The flexural strength of samples with a PhG content of 10% was: at 7 days of age, 4.92 MPa, which exceeds the strength of $M_{s}^{20}$ by 2.6% and the reference sample by 36.9%; at 14 days, the strength is 6.53 MPa, exceeding $M_{s}^{20}$ by 1.8% and the reference sample by 36.5%; at 28 days, it is 7.36 MPa, exceeding $M_{s}^{20}$ by 2.8% and the reference sample by 35.8%. Samples with a PhG content of 15% showed the following strength indicators: at 7 days, 4.99 MPa, which exceeds $M_{s}^{20}$ by 4.3% and the reference sample by 39.3%; at 14 days, the strength is 6.72 MPa, exceeding $M_{s}^{20}$ by 4.8% and the reference sample by 40.5%; at 28 days, it is 7.51 MPa, exceeding $M_{s}^{20}$ by 4.8% and the reference sample by 38.4%. For samples with a PhG content of 20%, the strength was: at 7 days, 4.93 MPa, which exceeds $M_{s}^{20}$ by 2.9% and the reference sample by 37.4%; at 14 days, the strength is 6.61 MPa, exceeding $M_{s}^{20}$ by 3.1% and the reference sample by 38.2%; at 28 days, it is 7.39 MPa, exceeding $M_{s}^{20}$ by 3.2% and the reference sample by 36.4%. For samples with a PhG content of 25%: at 7 days, the strength is 4.80 MPa, which is comparable to $M_{s}^{20}$ = 4.79 MPa, but 33.8% greater than the reference sample; at 14 days, the strength is 6.43 MPa, which is also comparable to $M_{s}^{20}$ = 6.41 MPa, but 34.6% greater than the reference sample; at 28 days, it is 7.30 MPa, which exceeds $M_{s}^{20}$ by 1.9% and the reference sample by 34.7%.

The coefficients of variation generally indicate a strong correlation between individual strength measurements and demonstrate a tendency toward stabilization as the samples gain strength. For compressive strength, the coefficients of variation ranged from 1.78% to 3.72%, while for flexural strength, they ranged from 1.36% to 2.98%. To verify the statistical significance of the observed improvements, a *t*-test was performed for the 28-day strength values. The compressive and flexural strength of mixtures containing 15% phosphogypsum were found to be significantly higher than those of the reference composition (*p* < 0.001).

**Figure 8:**
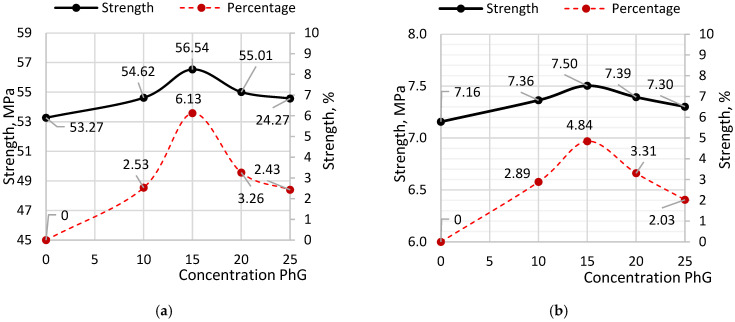
Change in strength based on the content of Phosphogypsum: (**a**) compressive strength; (**b**) flexural strength.

Figure 8 illustrates the variation in compressive strength as a function of phosphogypsum content in the samples.

According to the diagrams, the maximum increase in both compressive and flexural strength occurs at a phosphogypsum content of 15% relative to cement. Peak compressive strength reached 56.54 MPa, representing a 6.13% increase over samples without additives. Peak flexural strength reached 7.50 MPa, exceeding the reference sample by 4.84%. When phosphogypsum content exceeds 15%, a reduction in both compressive and flexural strength is observed. This decline may be associated with an excessive sulfate content introduced by phosphogypsum, leading to increased ettringite formation. An excessive amount of ettringite can disrupt the microstructure of the cement matrix and negatively affect strength development.

### 3.4. Results of Stage 3 Testing

**Figure 9:**
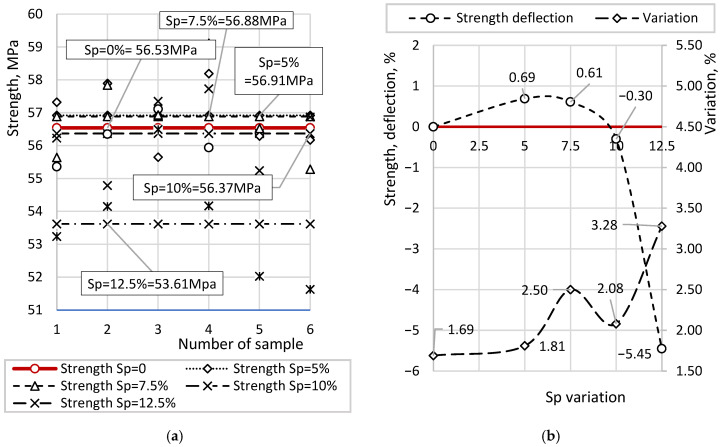
Compressive Strength Measurement of Samples: (**a**) individual and average values; (**b**) statistical indicators.

Since the flexural strength of beam samples was used as an additional evaluation criterion to assess the evolution of strength characteristics of the cement–sand mixture relative to previous stages, Figure 9 presents only the strength results measured at 28 days. Figure 9a shows the individual and average strength values at different concentrations of soapstock, while Figure 9b presents the statistical indicators of the coefficients of variation, along with a percentage comparison relative to the reference sample.

According to the measurement results, the individual strength values of the reference sample range from 55.56 to 58.39 MPa, with an average of 56.53 MPa. For samples with a soapstock content of Sp = 5%, the strength values vary from 55.65 to 58.19 MPa, with an average of 56.91 MPa. For samples with Sp = 7.5%, the strength ranges from 55.28 to 59.11 MPa, with an average of 56.88 MPa. Similarly, for Sp = 10%, the strength values lie between 54.79 and 57.35 MPa, with an average of 56.37 MPa. For samples containing Sp = 12.5%, the strength ranges from 51.63 to 56.49 MPa, with an average of 56.61 MPa.

For samples with soapstock contents of 5%, 7.5%, and 10%, the strength values are comparable to those of the reference sample. According to the curves in Figure 9b, the differences are 0.69%, 0.61%, and −0.30%, respectively. In contrast, samples with a soapstock content of 12.5% show a noticeable deviation from the reference sample, with a difference of −5.45%. This indicates a negative effect of soapstock at higher concentrations in the concrete mixture.

The analysis of the coefficients of variation suggests that a high soapstock content reduces the structural stability of the concrete, as evidenced by the increased scatter of individual strength values. Therefore, no significant positive effect of soapstock on concrete strength was observed; moreover, its high concentration adversely affects strength.

**Figure 10:**
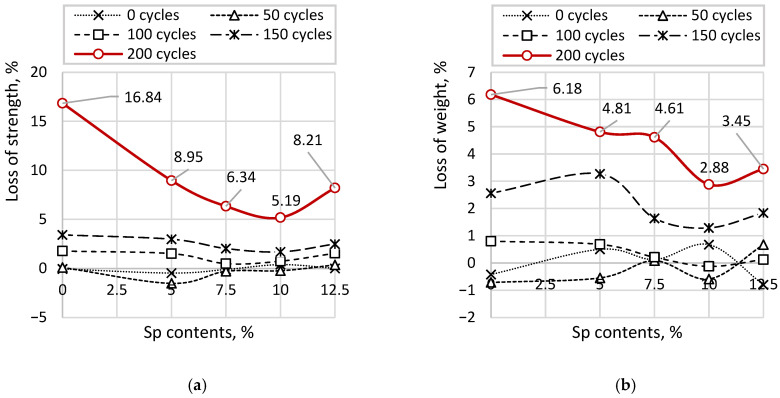
Strength and Mass Loss under Cyclic Freeze–Thaw Conditions: (**a**) values of strength loss; (**b**) values of mass loss.

Figure 10 presents the results of strength loss and mass loss due to cyclic freeze–thaw exposure of cubic samples with varying soapstock (Sp) content. Figure 10a shows the absolute individual values of strength loss, while Figure 10b illustrates the mass loss. The red solid line, corresponding to the maximum strength and mass losses after 200 cycles, indicates the percentage values relative to the reference sample.

The strength of the reference sample over freeze–thaw cycles from 50 to 200 decreases from 62.32 to 55.20 MPa. The maximum reduction in strength is observed at the highest number of cycles, while a noticeable decline begins after 100 cycles. At the same time, the coefficients of variation increase with the number of cycles, ranging from 2.45% to 12.78%. For samples with the lowest soapstock content (Sp = 5.0%), the strength varies from 65.38 to 60.44 MPa, with a decline also beginning after 100 cycles. The coefficients of variation follow a similar trend to those of the reference sample, ranging from 3.03% to 8.75%. For samples with Sp = 7.5%, the strength ranges from 66.45 to 62.17 MPa, with a noticeable decrease occurring from 125 cycles onward. The coefficients of variation increase from 1.53% to 6.82%. For samples with Sp = 10.0%, a significant reduction in strength is observed starting from 150 cycles, with strength values ranging from 66.12 to 62.94 MPa and coefficients of variation from 3.41% to 5.69%. For samples with the highest soapstock content (Sp = 12.5%), the reduction in strength begins at 125 cycles, with values decreasing from 63.33 to 58.13 MPa and coefficients of variation ranging from 2.31% to 7.83%. However, these samples exhibit an overall reduction in strength compared to the reference sample, averaging 4.8%, which indicates a negative effect of high soapstock content on the mechanical properties of concrete. The mass loss curves generally follow trends similar to those observed for strength. For the reference sample, the mass decreases from 2388 to 2231 g, with the onset of noticeable loss occurring at 100 cycles. As with strength, increasing the number of cycles leads to higher coefficients of variation (for all Sp contents), ranging from 1.36% to 9.23%. For samples with Sp = 5.0%, mass loss varies from 2366 to 2264 g, with the initial decrease also occurring at 100 cycles, while the coefficients of variation increase from 1.36% to 7.12%. Samples with Sp = 7.5% show mass loss from 2375 to 2268 g, with noticeable losses beginning at 125 cycles and coefficients of variation increasing from 1.36% to 6.42%. For samples with Sp = 10.0%, mass loss begins at 150 cycles, ranging from 2362 to 2309 g, while the coefficients of variation vary from 1.36% to 4.54%. For samples with the highest soapstock content (Sp = 12.5%), mass loss ranges from 2397 to 2296 g, with coefficients of variation between 1.36% and 6.39%. The onset of mass loss is observed at both 50 and 125 cycles, whereas the losses at 100 cycles are minimal and comparable to those of the reference sample. Such inconsistency indicates a negative effect of high soapstock concentrations on the quality and durability of concrete.

The analysis of the coefficients of variation in both cases indicates a decrease in the stability of strength (and mass) results with an increasing number of freeze–thaw cycles; however, their quantitative values depend on the relative changes in strength (and mass) compared to the initial state (corresponding to 0 cycles). This is confirmed by the lowest coefficients of variation observed in samples with Sp = 10.0%, which also exhibit the smallest strength loss. Quantitatively, the coefficient of variation for strength in samples with Sp = 10.0% is 2.24 times lower than that of the reference sample and 1.11–1.53 times lower compared to other Sp variations. Similarly, the coefficient of variation for mass in these samples is 2.14 times lower than that of the reference sample and 1.17–1.67 times lower than in other Sp variations.

The average maximum strength loss of the reference sample is 16.84%, while the average mass loss is 6.18%. For samples with Sp = 5%, the maximum strength loss is 8.95% and the maximum mass loss is 4.81%. For Sp = 7.5%, these values are 6.34% (strength) and 4.61% (mass). For Sp = 10.0%, the corresponding losses are 5.69% and 2.88%, respectively. For samples with the highest soapstock content (Sp = 12.5%), the maximum strength loss is 7.83% and the mass loss is 3.45%. Thus, the highest resistance to cyclic freeze–thaw action is observed in samples with Sp = 10.0%. In terms of strength and mass loss, the frost resistance increases by approximately 50% (150/100) compared to the reference sample, and by 20% to 50% (125/100–150/100) compared to other Sp variations.

**Figure 11:**
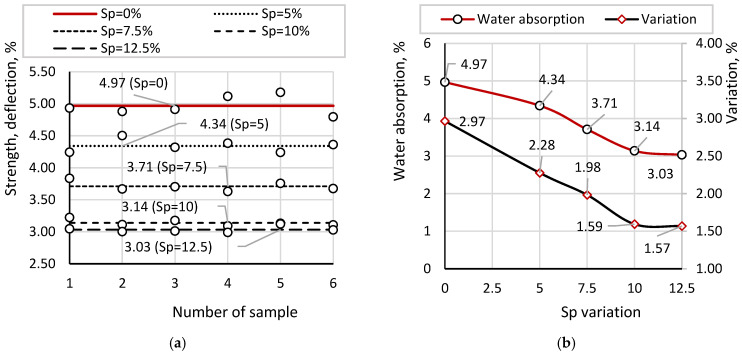
Water Absorption Results: (**a**) individual water absorption values; (**b**) average water absorption values.

Figure 11 presents the results of water absorption measurements for samples with different Sp contents. Figure 11a shows the individual water absorption values along with their corresponding averages. The horizontal lines in the diagram represent the mean water absorption for each sample type, facilitating visualization of deviations of individual values from the mean. Figure 11b compares the average water absorption values for samples with different Sp contents, along with their corresponding coefficients of variation.

According to the results, the average value of six water absorption measurements for the reference sample is 4.97%, with values ranging from 4.79% to 5.18%. For samples with Sp = 5%, water absorption varies from 4.24% to 4.51%, with an average of 4.34%. For Sp = 7.5%, the values range from 3.63% to 3.83%, with an average of 3.71%. For Sp = 10%, water absorption varies from 3.09% to 3.22%, with an average of 3.14%. For Sp = 12.5%, the values range from 2.99% to 3.12%, with an average of 3.03%.

An increase in soapstock content consistently leads to a reduction in water absorption. Compared to the reference sample, the reduction is 12.6% for Sp = 5%, 25.3% for Sp = 7.5%, 36.8% for Sp = 10%, and 39.0% for Sp = 12.5%.

However, the decrease in water absorption with respect to the linear increase in soapstock content (in increments of 2.5%) is non-linear. At an up to 10% soapstock content, a pronounced reduction in water absorption is observed, followed by a significantly smaller decrease. Quantitatively, the incremental reductions are 12.6%, 14.5%, 15.4%, and 3.4% for each successive 2.5% addition of soapstock. The reduced efficiency at higher concentrations indicates a diminishing hydrophobic effect of soapstock at elevated dosages.

The analysis of the coefficients of variation demonstrates a high degree of consistency among individual measurements for all compositions, including the reference sample, with values ranging from 1.57% to 2.97%. The scatter of results decreases with increasing soapstock content, indicating improved stability, which corresponds to enhanced hydrophobization of the samples.

To assess the reliability of the obtained results, a statistical analysis was performed. The observed differences in compressive strength, frost resistance, and water absorption between the compositions were found to be statistically significant (*p* < 0.05), confirming the consistency of the identified trends.

### 3.5. Results of Stage 4 Testing

**Figure 12:**
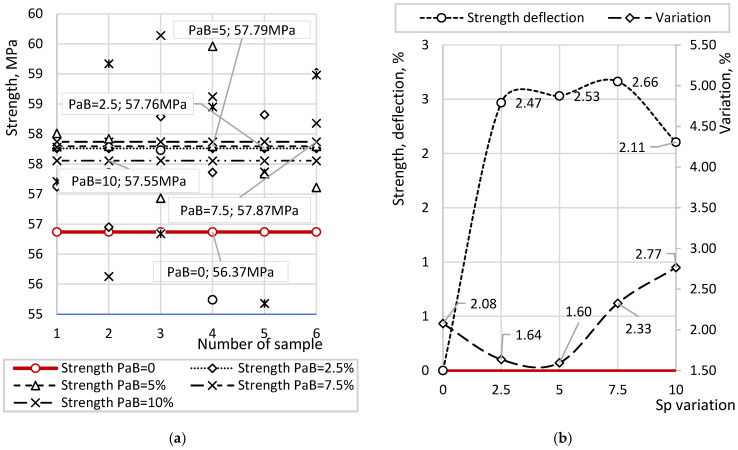
Compressive Strength Measurement of Samples: (**a**) individual and average values; (**b**) statistical indicators.

Figure 12 presents the results of compressive strength measurements of beam samples, obtained as part of a stepwise evaluation of sequential additions of the components Ms, PhG, Sp, and PaB. Figure 12a shows individual and average strength values at different concentrations of PaB. In contrast, Figure 12b presents the corresponding coefficients of variation and percentage comparisons relative to the optimal mixture containing Ms = 20%, PhG = 15%, and Sp = 10%.

Based on previous tests, the optimal concentration of Sp (10%) corresponded to an average compressive strength of 56.37 MPa. With the addition of PaB at 2.5%, the average strength increased to 57.76 MPa, with individual values ranging from 55.45 to 59.02 MPa. For PaB at 5%, strength ranged from 56.93 to 59.46 MPa, with an average of 57.79 MPa; at 7.5%, the range was 55.63–59.64 MPa, with an average of 57.87 MPa; and at 10%, strength ranged from 55.18 to 59.17 MPa, averaging 57.55 MPa.

According to the comparative diagram in Figure 12b, no significant changes in strength were observed as PaB concentration increased. Noticeable improvement occurred only with the initial addition of 2.5% PaB relative to the water content. Variance analysis indicated no substantial impact of PaB on result stability, with coefficients of variation ranging from 1.60% to 2.77%, indicating high consistency among individual measurements. The lack of effect of PaB at concentrations above 2.5% is further confirmed by the variation coefficient of the average strength across all PaB concentrations, which was only 0.23%. Compared to the variation within a single concentration (1.60–2.77%), changes in strength due to varying PaB concentrations fall within the statistical error range. Therefore, the optimal concentration of PaB is 2.5%, as higher concentrations do not provide additional strength.

**Figure 13:**
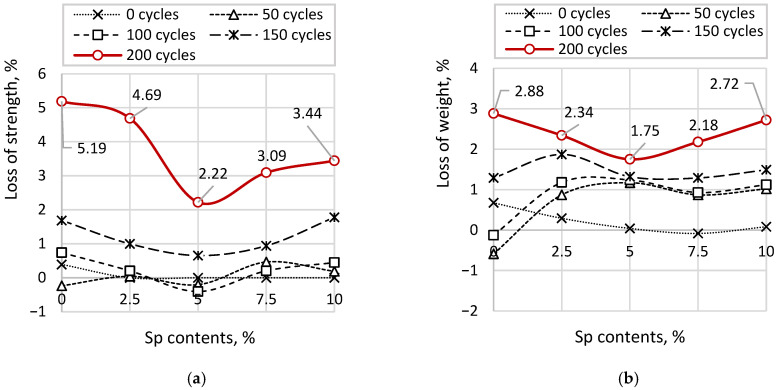
Strength and Mass Loss during Cyclic Freezing: (**a**) loss of strength; (**b**) loss of weight.

Figure 13 presents the graphs of compressive strength and mass losses of cubic samples subjected to cyclic freezing and thawing with varying levels of post-alcohol bard (PaB) addition. Figure 13a shows the absolute individual strength loss values, while Figure 13b illustrates mass loss.

For samples containing 2.5% PaB, compressive strength decreased from 69.12 MPa at 50 cycles to 65.19 MPa at 200 cycles. The maximum strength reduction corresponds to the highest number of cycles, with a noticeable decline beginning around cycles 150–175. The coefficients of variation increased with the number of cycles, ranging from 1.93% to 4.85%.

Samples with 5.0% PaB showed strength reductions from 68.88 to 67.35 MPa, with the decrease starting between cycles 175 and 200. Coefficients of variation remained relatively stable, ranging from 1.77% to 3.32%. For samples with 7.5% PaB, strength ranged from 69.17 to 67.03 MPa, with a noticeable decline beginning around the 175th cycle and coefficients of variation ranging from 3.41% to 5.69%. Samples with the highest PaB content (10.0%) displayed a similar reduction pattern starting at 175 cycles, with strength values from 69.15 to 66.77 MPa and coefficients of variation between 2.31% and 7.83%.

The mass loss trends closely mirror the strength reduction patterns. For samples with 2.5% PaB, mass decreased from 2386 g to 2310 g, with initial losses observed at 100 cycles, and coefficients of variation rising from 1.36% to 7.12%. Samples with 5.0% PaB exhibited mass losses from 2392 to 2344 g, with the initial decrease at 150 cycles and coefficients of variation increasing from 1.36% to 2.98%. Samples with 7.5% PaB showed mass losses from 2395 to 2318 g, with initial reduction around 150 cycles and coefficients of variation increasing from 1.36% to 4.54%. Samples containing 10.0% PaB began to lose mass at 150 cycles, with values ranging from 2391 to 2321 g and coefficients of variation from 1.36% to 4.39%.

It should be noted that the initial strength values for all PaB concentrations are comparable within the range of statistical error. This indicates that samples with different PaB contents exhibited a similar strength increase relative to the reference sample. The magnitude of strength gain is largely independent of PaB concentration: for PaB = 2.5%, the average increase was 4.12%; for PaB = 5.0%, 3.77%; for PaB = 7.5%, 4.20%; and for PaB = 10.0%, 4.17%. Similar increases were observed in samples with an optimal Sp content of 10%, ranging from 4.17% to 4.61%. These minor deviations can be attributed to statistical error, as they do not exceed the previously determined variation in the reference sample (1.45%).

Strength reduction for all PaB concentrations, except 2.5%, begins around cycle 175, although the rate of decrease varies: higher PaB contents lead to a more pronounced strength loss beyond 175 cycles. Analysis of the coefficients of variation indicates a decline in the stability of both strength and mass results with increasing freezing cycles. However, the magnitude of this decline depends on the extent of strength and mass loss relative to the initial values (at 0 cycles).

This is further evidenced by the samples containing 5.0% PaB, which experienced the least strength loss at 200 cycles. Quantitatively, the strength variation coefficient for these samples was 3.09 times lower than that of the reference sample and 1.29–2.03 times lower than for the other PaB concentrations. Similarly, the mass variation coefficient for 5.0% PaB samples was 3.85 times lower than the reference and 1.63–2.64 times lower than other PaB variations. Overall, variation analysis demonstrates that samples with 5.0% PaB exhibited the most stable performance at 200 cycles in terms of both strength and mass.

The curves in Figure 13 clearly illustrate the influence of post-alcohol bard (PaB) on the durability of concrete, specifically regarding its resistance to cyclic freezing. For samples containing 2.5% PaB, the maximum strength loss was 4.69%, and the maximum mass loss was 2.34%, which are 2.9 and 1.9 times lower, respectively, than the corresponding values for the reference sample. For samples with 5% PaB, these losses were further reduced, with strength loss of 2.22% and mass loss of 1.75%, representing reductions of 7.6 and 3.5 times compared to the reference sample, and 2.3 and 1.6 times lower than samples with an optimal Sp content of 10%. Samples with 7.5% PaB exhibited strength and mass losses of 3.09% and 2.86%, respectively, which are 5.4 and 2.2 times lower than the reference values. For the highest PaB concentration of 10%, strength loss was 3.44% and mass loss 2.72%, corresponding to reductions of 4.9 and 2.3 times relative to the reference sample.

Therefore, the highest resistance to cyclic freezing was observed in samples containing 5% PaB. At this concentration, frost resistance in terms of strength increased by 100% (200/100) relative to the reference sample and by 33% (200/150) compared to samples with an optimal Sp content of 10%. For other PaB concentrations, frost resistance in terms of strength increased by approximately 75% (175/100) relative to the reference sample. Regarding mass loss, frost resistance at 5% PaB increased by 33% (200/150) relative to the reference sample and by 14% (200/175) compared to samples with Sp = 10%. For the remaining PaB concentrations, frost resistance in terms of mass increased by 17% (175/200) relative to the reference sample.

**Figure 14:**
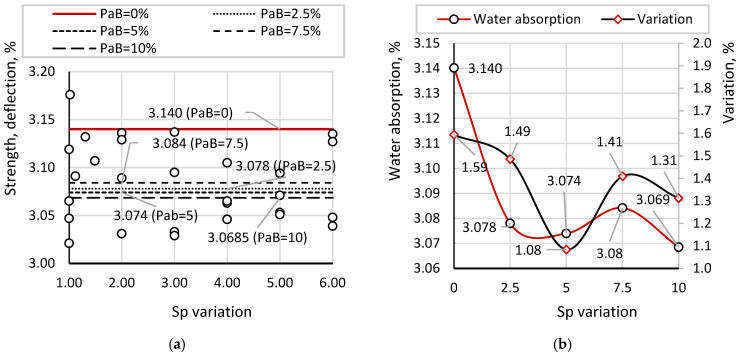
Water Absorption Results: (**a**) individual and average values; (**b**) statistical indicators.

Figure 14 presents the water absorption results for concrete samples with varying PaB contents, all incorporating the previously determined optimal Sp concentration of 10%. In Figure 14a, both individual and average water absorption values are shown, with straight lines representing the average for each sample type to visualize deviations. Figure 14b provides a comparison of average water absorption values across samples with different PaB concentrations, together with their respective coefficients of variation.

According to previous tests, the average water absorption for samples with Sp = 10% was 3.150%. This value serves as a reference for evaluating the effectiveness of PaB and for determining its optimal concentration.

For samples with PaB = 2.5%, water absorption ranged from 3.033% to 3.136%, with an average of 3.078%. For PaB = 5%, the values varied between 3.039% and 3.129%, averaging 3.074%. Samples with PaB = 7.5% showed water absorption from 3.031% to 3.127%, with an average of 3.084%, while for PaB = 10%, the range was 3.021% to 3.137%, with an average of 3.069%. The addition of a minimal amount of bard resulted in a reduction in water absorption; however, further increases in PaB concentration did not produce significant changes.

Compared to samples with Sp = 10% and PaB = 0%, the reductions in water absorption were as follows: 2.29% for PaB = 2.5%, 2.47% for PaB = 5%, 2.18% for PaB = 7.5%, and 2.66% for PaB = 10%. Incremental changes in water absorption with each subsequent 2.5% addition of bard were 0.13%, −0.29%, and 0.49%, respectively. The pronounced reduction in water absorption with the initial bard addition, followed by stabilization at higher concentrations, indicates that the hydrophobizing effect reaches its maximum efficiency with minimal PaB content.

Analysis of the coefficients of variation demonstrated a high degree of consistency among individual values across all PaB concentrations, ranging from 1.31% to 1.59%. Statistical evaluation of the average water absorption values confirmed that increasing PaB content does not significantly affect water absorption, with the coefficient of variation for the average values being only 0.99%. Considering the variation within each sample type (1.31% to 1.59%), the 0.99% coefficient for averages suggests that observed differences in water absorption at varying PaB concentrations can be attributed to statistical error. The observed differences in compressive strength, frost resistance, and water absorption between the compositions were found to be statistically significant (*p* < 0.05), confirming the consistency of the identified trends.

### 3.6. Summary of Research Results

The results of the experimental studies are summarized in a table illustrating the effects of the sequential addition of the modified additive components on the primary physical and mechanical properties of concrete. The Table 4 consolidates the outcomes for the optimal compositions determined at each stage of the study, including compressive and flexural strength, water absorption, and frost resistance. The optimal component proportions were identified based on a combination of criteria, encompassing strength, water absorption, and frost resistance.

The presented results demonstrate a consistent improvement in the performance characteristics of concrete with the stepwise addition of the components of the modified additive.

The incorporation of microsilica at 20% of the cement mass resulted in a significant increase in the strength of the cement–sand mixture, with compressive strength reaching 53.27 MPa, 28.3% higher than that of the control samples. A similar trend was observed for flexural strength.

The subsequent addition of phosphogypsum at 15% of the binder mass further enhanced strength, achieving 56.54 MPa in compression and 7.50 MPa in flexure. This improvement is attributed to changes in the mineralogical composition of the cement paste and the optimized interaction between calcium and silica compounds.

The inclusion of soapstock had a minimal effect on strength; however, it significantly improved the operational properties of the concrete. Notably, water absorption decreased from 4.97% to 3.14%, indicating a pronounced hydrophobizing effect of the additive. Concurrently, frost resistance was markedly enhanced, as evidenced by reduced strength and mass losses during cyclic freezing.

The addition of post-alcohol bard moderately influenced the strength characteristics but substantially increased the durability of the material. The most notable effect was observed at a concentration of 5% of the mixing water mass, where strength loss after 200 freeze–thaw cycles was only 2.22%, and mass loss was 1.75%.

Overall, these results confirm the effectiveness of the developed dual-component additive based on industrial waste, which simultaneously enhances strength, reduces water absorption, and significantly improves the frost resistance of concrete.

## 4. Discussion

The EDX results revealed changes in the elemental composition of the cement matrix with the sequential addition of the components of the modifying additive. Variations in the content of key elements (Si, Ca, Al, S, and O) reflect transformations in cement hydration processes and the development of the cement composite structure. The EDX analysis of the control sample indicated a relatively high oxygen content (~57%), which may suggest the presence of oxide compounds not actively involved in the formation of hydrate phases.

With the introduction of microsilica, an increase in silicon content and a decrease in oxygen content to approximately 40% at a concentration of Ms = 20% were observed. This change is associated with the pozzolanic reaction of SiO_2_ with portlandite, resulting in the formation of additional calcium silicate hydrates (C–S–H) and densification of the cement matrix [38,39,40]. Similar effects of structural densification and strength enhancement in concrete using microsilica have been reported in previous studies on mineral additives in high-strength cement composites [41]. A further increase in microsilica content to 25% caused a rise in oxygen content, which may indicate a decrease in the efficiency of the pozzolanic reaction due to limited availability of portlandite or the need for a longer time for the reaction to proceed to completion [42]. The addition of phosphogypsum resulted in increased calcium and sulfur contents, attributable to its high CaSO_4_ composition, which may facilitate the formation of sulfoaluminate phases, including ettringite [43]. The introduction of soapstock and post-alcohol bard did not significantly alter the content of the primary elements (Ca and Si) in the cement binder; however, these components influenced the alkalinity of the medium and the development of the cement matrix structure. Overall, the EDX analysis confirms that the sequential addition of the additive components induces measurable changes in the chemical composition and microstructure of the cement composite, consistent with the observed improvements in mechanical and performance properties of the concrete. Recent studies on multi-component cementitious systems also report that synergistic effects arise from the combined action of chemical interactions and microstructural densification. For example, magnesium cement–fly ash composites demonstrate enhanced strength and durability due to coupled hydration reactions and pore refinement [44,45].

These microstructural changes provide a basis for interpreting the strength behaviour of the modified system. The study of the effect of phosphogypsum on concrete strength also revealed a peak-type relationship, indicating the existence of an optimal additive content. The observed decrease in strength at phosphogypsum contents above 15% may be associated with a disruption in the balance between calcium-containing and sulfate phases in the cement binder. The introduction of phosphogypsum in moderate amounts contributes to the regulation of cement hydration processes and the formation of sulfoaluminate compounds, primarily ettringite, which participate in the development of the cement matrix at early stages of hardening. The formation of these phases may lead to densification of the cement paste microstructure and improvements in the material’s strength characteristics. However, at higher concentrations of calcium sulfate, excessive sulfate phases may form, potentially disturbing the stability of hydrate compounds and slowing the strength development of the cement matrix at later stages. Specifically, an increase in sulfate compounds may alter the composition of hydration products, resulting in the formation of ettringite and AFm phases, which can affect pore structure and mechanical properties. The main hydration products of cement systems remain calcium silicate hydrates (C–S–H), which primarily determine the strength of the cement matrix [46]. These results are consistent with previously published studies, indicating that phosphogypsum, whose main component is CaSO_4_·2H_2_O, can serve as a source of calcium sulfate and a regulator of hydration processes in cement systems. However, excessive phosphogypsum content may adversely affect the structure and strength of cement composites [47,48,49]. Similar behaviour has been reported in composite systems where the balance between sulfate phases and binding components governs the overall performance of the material [50]. It should be noted that the selection of PhG = 15% as the optimal content is based not only on the maximum strength values, but also on the observed stability of the results and the consistent decrease in strength at higher concentrations. This indicates the presence of an optimal balance between sulfate content and cement hydration processes. Excessive phosphogypsum leads to an imbalance in sulfate phases, negatively affecting the long-term development of the cement matrix.

The next stage of the experimental program focused on assessing the durability and frost resistance of concrete modified with soapstock. Unlike the previous stages, this phase emphasized the effects of cyclic temperature exposure and water saturation on the long-term performance of the cement composite. The results obtained are generally consistent with previous studies investigating the influence of hydrophobic additives on the durability of cement-based materials [51]. It was found that the effect of the soapstock solution on compressive strength is limited, with the maximum positive effect observed at a content of approximately Sp ≈ 5%. At the same time, frost resistance gradually increased with higher soapstock concentrations, likely due to the hydrophobization of the cement matrix and the resulting reduction in capillary water absorption. As noted in several studies, hydrophobic organic compounds can reduce the surface energy of pores, limiting water penetration into the pore structure of the cement matrix, thereby enhancing the resistance of concrete to cyclic freezing and thawing [38,48]. However, despite a slight increase in hydrophobicity at Sp = 12.5% compared with Sp = 10%, frost resistance decreased. This phenomenon may be explained by a reduction in the structural integrity of the cement matrix caused by an excess of fatty acids in the soapstock solution. During neutralization of soapstock with NaOH, the fatty acids become water-soluble and are evenly distributed within the cement paste. During hydration, these compounds can form hydrophobic films on the surfaces of pores and capillaries. However, at high concentrations, such compounds may partially inhibit hydration reactions and reduce the adhesion between hydration products and aggregates, negatively affecting both strength and durability [52]. Analysis of the relationship between water absorption and soapstock concentration revealed that the greatest reduction in water absorption occurs at Sp = 10%. Further increases in soapstock content led only to minor additional decreases, suggesting that the maximum hydrophobization of the cement matrix had already been achieved.

The study of the effect of post-alcohol bard on concrete strength showed that the maximum increase in strength occurs at a relatively low additive concentration of approximately 2.5%. It should be noted that the onset of strength reduction and mass loss during cyclic freeze–thaw exposure occurred at roughly the same number of cycles for all PaB concentrations (about 175 cycles for strength and 150 cycles for mass). However, at later stages of cyclic exposure, higher PaB concentrations resulted in a more pronounced decrease in strength. Evaluation of compressive strength and water absorption indicates that the optimal PaB concentration for improving strength is around 2.5%, whereas the best frost-resistance performance is observed at PaB ≈ 5%. This discrepancy may be related to changes in the internal stress state of the cement matrix during cyclic water saturation, freezing, and thawing. Organic compounds present in biochemical by-products are known to exhibit a plasticizing effect, altering the pore structure of cement composites, contributing to the formation of a denser microstructure, and reducing capillary water absorption [38,53]. At elevated PaB concentrations, the number of micropores in the binder structure may decrease, reducing the contact area between the cement matrix and inert aggregates, potentially resulting in a more brittle material. Nevertheless, until partial structural degradation occurs, the denser microstructure and the plasticizing effect of the organic components of PaB enhance the material’s resistance to cyclic freeze–thaw action. Analysis of the relationship between water absorption and PaB concentration confirms that the most effective reduction in water absorption is achieved at PaB ≈ 2.5%, with further increases in additive content producing no significant additional decrease. These patterns are consistent with previously reported studies on the influence of organic additives on the structure and durability of cement-based materials [7,9,10,20,22,28,29,30,54,55]. It should be emphasized that the effect of PaB on compressive strength is relatively limited. The observed increase in strength at 2.5% is within 2–4%, which is comparable to the experimental variability. Therefore, PaB is not considered as a strength-enhancing component. Its primary role is associated with modification of durability-related properties, including reduction in water absorption and improvement of freeze–thaw resistance.

In addition to the individual effects of each component, the combined action of the four-component system demonstrates a pronounced synergistic effect. The mineral components (microsilica and phosphogypsum) primarily contribute to the formation and densification of the cement matrix through pozzolanic reactions and regulation of hydration processes. This results in a refined pore structure and improved mechanical performance. At the same time, the organic components (soapstock and post-alcohol bard) modify the pore system and interfacial properties of the cement composite. Soapstock provides hydrophobization and reduces capillary water absorption, while post-alcohol bard exhibits a plasticizing effect, improving the distribution of particles and facilitating the formation of a more homogeneous microstructure. The interaction between these components can be considered synergistic rather than antagonistic. The densified matrix formed by mineral additives enhances the effectiveness of hydrophobic modification, while the plasticizing effect of post-alcohol bard improves the dispersion of both mineral and organic components within the system. As a result, the combined system enables simultaneous improvement of strength, durability, and water resistance, which cannot be achieved by individual components acting separately. These findings are consistent with recent studies on multi-component stabilizing systems, where synergistic effects are attributed to the interaction between mineral and organic phases, leading to improved mechanical performance and durability of cement-based materials [44,45,50].

The optimal formulation identified in this study is based on the specific materials used and may vary with changes in cement composition or aggregate characteristics. Therefore, its applicability to other material systems requires further validation.

It should be noted that the present study focuses on early and medium-term properties, including 28-day strength, water absorption, and freeze–thaw resistance. Long-term strength development (90 and 180 days) and additional durability indicators such as carbonation resistance and chloride ion penetration were not considered and represent a limitation of the current work. These aspects will be addressed in future studies.

## 5. Conclusions

The conducted experimental studies aimed to determine the optimal composition of a four-component modifying additive based on industrial waste and to evaluate its influence on the strength, durability, and operational characteristics of concrete. The experimental program was organized in a staged manner, allowing the individual effects of each additive component to be assessed and optimal concentrations to be identified.

1. EDX Analysis: The analysis revealed a natural increase in the content of chemical elements introduced with the additives into the cement paste: the addition of microsilica (Ms) increased Si content, phosphogypsum (PhG) increased Ca, and the introduction of soapstock (Sp) and post-alcohol bard (PaB) increased Al and S. Changes in the elemental composition of the cement paste contributed to transformations in the microstructure of the binder, subsequently affecting the physical and mechanical properties of concrete.

2. Stage 1—Effect of Microsilica (Ms): Compressive strength tests showed that the maximum strength was achieved with Ms = 20%, while the lowest values were observed in the reference sample. The average increase in compressive strength relative to the reference sample was 9.7% for Ms = 10%, 18.1% for Ms = 15%, 28.3% for Ms = 20%, and 24.8% for Ms = 25%. A similar trend was observed for flexural strength. When the microsilica content exceeded 20%, a slight decrease in strength was noted, indicating the existence of an optimal dosage.

3. Stage 2—Effect of Phosphogypsum (PhG): Compressive strength tests indicated that the maximum strength increase occurred at PhG = 15%. The average increase relative to the reference sample was 2.5% for PhG = 10%, 6.1% for PhG = 15%, 3.3% for PhG = 20%, and 2.4% for PhG = 25%. Flexural strength tests showed a similar pattern. Increasing the phosphogypsum content above 15% resulted in a decrease in strength, suggesting that excessive sulfate content negatively affects the structural stability of the cement matrix.

4. Stage 3—Effect of Soapstock (Sp): Strength tests indicated that the incorporation of soapstock in the range of 5–10% did not lead to a significant increase in compressive strength compared to the reference sample, while a higher content (Sp = 12.5%) resulted in a noticeable strength reduction of up to 5.45%, indicating a negative effect at elevated dosages. Frost-resistance tests showed that the highest resistance to cyclic freeze–thaw action was achieved at Sp = 10.0%, where the lowest strength and mass losses were observed. At this concentration, frost resistance improved by up to 50% compared to the reference sample, while both lower and higher Sp contents demonstrated reduced performance. Water absorption tests revealed a consistent decrease with increasing soapstock content, with the most significant reduction observed at Sp = 10–12.5% (up to 39% compared to the reference sample). However, the rate of improvement diminished at higher concentrations, indicating a reduced efficiency of the hydrophobic effect.

5. Stage 4—Effect of Post-alcohol Bard (PaB): Strength tests showed that the optimum increase in strength occurred at PaB = 2.5%, while further increases in bard content did not result in significant strength changes. The average increase in compressive strength at PaB = 2.5% was approximately 2.5%. Water absorption tests indicated the most pronounced reduction at the same concentration, with an average decrease of about 2.3%. Frost-resistance tests demonstrated that the highest resistance to cyclic freezing and thawing was achieved at PaB = 5%, while further increases in bard concentration reduced frost resistance.

Based on these results, the optimal composition of the developed modifying additive was determined. The best combination of strength, frost resistance, and reduced water absorption was achieved with the following proportions: microsilica Ms = 20% by weight of cement, phosphogypsum PhG = 15% by weight of cement and microsilica replacement, soapstock Sp = 10% by weight of cement, microsilica, and phosphogypsum replacement, and post-alcohol bard PaB = 5% by weight of mixing water. Quantitative evidence demonstrates the additive’s potential for sustainable development: cement replacement with microsilica and phosphogypsum reached up to 35% of the binder mass, compressive strength increased by up to 28.3%, water absorption decreased by 36.8–39%, and freeze–thaw resistance improved by up to 50% relative to the reference. The results demonstrate the potential of using industrial waste in the composition of modifying additives for concrete and confirm the feasibility of enhancing operational performance while promoting resource conservation and sustainable development in the construction industry.

## Figures and Tables

**Table 1:** Variable Compositions of the Studied Mixtures [28].

Stages	Component Mass Content, g.							
	Sand	Cement	Ms	PhG	Sp	NaOH	PaB	Water
Reference	1500	500	0	-	-	-	-	200
Stage 1	1500	450	50	-	-	-	-	200
	1500	425	75	-	-	-	-	200
	1500	400	100	-	-	-	-	200
	1500	375	125	-	-	-	-	200
Stage 2	1500	450		50	-	-	-	200
	1500	425		75	-	-	-	200
	1500	400		100	-	-	-	200
	1500	375		125	-	-	-	200
Stage 3	1500	475.0			24.750	0.250	-	200
	1500	462.5			37.125	0.375	-	200
	1500	450.0			49.500	0.500	-	200
	1500	437.5			61.875	0.625	-	200
Stage 4	1500	500					5	195
	1500	500					10	190
	1500	500					15	185
	1500	500					20	180

**Table 2:** Chemical composition of cement [30].

Parameters	Content (% by Weight)									
	Na_2_O	MgO	Al_2_O_3_	SiO_2_	SO_3_	K_2_O	CaO	TiO_2_	MnO	FeO
Average	0.12	1.05	3.80	21.60	3.39	0.80	65.18	0.25	0.24	3.56
Standard. deviation	0.16	0.08	0.30	0.44	0.08	0.10	0.27	0.13	0.15	0.14
Max.	0.23	1.14	4.07	21.91	3.44	0.90	65.48	0.34	0.42	3.65
Min.	−0.05	1.00	3.47	21.10	3.30	0.72	64.95	0.11	0.12	3.39

**Table 3:** Physical and Chemical Characteristics of Additive Components.

No.	Additive Component	Main Chemical Composition	Particle Size	pH Range
1	Microsilica (Ms)	>92% amorphous SiO_2_	0.1–0.3 µm	4.5–6.5
2	Phosphogypsum (PhG)	>90% CaSO_4_·2H_2_O	10–100 µm	6.5–7.5
3	Soapstock (Sp)	Fatty acids, phospholipids, triglycerides	<50 µm	9.5–10.5
4	Post-alcohol bard (PaB)	Organic acids, proteins (casein)	<100 µm	4.0–5.5

**Table 4:** Summary of the Research Results.

Composition	Additive Content, %				A	B	C	D	E
	Ms	PhG	Sp	PaB					
Reference	0	0	0	0	41.53	5.42	4.97	16.84	6.18
Ms	20	0	0	0	53.27	7.14	-	-	-
Ms + PhG	20	15	0	0	56.54	7.5	-	-	-
Ms + PhG + Sp	20	15	10	0	56.37	-	3.14	5.69	2.88
Ms + PhG + Sp + PaB	20	15	10	2.5	57.76	-	3.08	4.69	2.34
Ms + PhG + Sp + PaB	20	15	10	5	57.79	-	3.07	2.22	1.75

A—compressive strength, MPa; B—flexural strength, MPa; C—water absorption, %; D—strength loss after 200 cycles, %; E—mass loss after 200 cycles, %.

## Data Availability

The original contributions presented in this study are included in the article. Further inquiries can be directed to the corresponding author.
